# Anti-inflammatory effects of *Phyllanthus amarus* Schum. & Thonn. through inhibition of NF-κB, MAPK, and PI3K-Akt signaling pathways in LPS-induced human macrophages

**DOI:** 10.1186/s12906-018-2289-3

**Published:** 2018-07-25

**Authors:** Hemavathy Harikrishnan, Ibrahim Jantan, Md. Areeful Haque, Endang Kumolosasi

**Affiliations:** 1 Drug and Herbal Research Center, Faculty of Pharmacy, Universiti Kebangsaan Malaysia, Jalan Raja Muda Abdul Aziz, 50300 Kuala Lumpur, Malaysia; 2 School of Pharmacy, Taylor’s University, Lakeside Campus, 47500 Subang Jaya, Selangor Malaysia

**Keywords:** *Phyllanthus amarus*, Macrophages, Inflammation, Cytokines, NF-κB, MAPK, PI3K-Akt

## Abstract

**Background:**

*Phyllanthus amarus* has been used widely in various traditional medicines to treat swelling, sores, jaundice, inflammatory diseases, kidney disorders, diabetes and viral hepatitis, while its pharmacological and biochemical mechanisms underlying its anti-inflammatory properties have not been well investigated. The present study was carried out to investigate the effects of 80% ethanolic extract of *P. amarus* on pro-inflammatory mediators release in nuclear factor-kappa B (NF-кB), mitogen activated protein kinase (MAPK) and phosphatidylinositol 3-kinase/Akt (PI3K-Akt) signaling activation in lipopolysaccharide (LPS)-induced U937 human macrophages.

**Methods:**

The release of prostaglandin E_2_ (PGE_2_) and pro-inflammatory cytokines, tumor necrosis factor (TNF)-α and interleukin (IL)-1β in a culture supernatant was determined by ELISA. Determination of cyclooxygenase-2 (COX-2) protein and the activation of MAPKs molecules (JNK, ERK and p38 MAPK), NF-κB and Akt in LPS-induced U937 human macrophages were investigated by immunoblot technique. The relative gene expression levels of COX-2 and pro-inflammatory cytokines were measured by using qRT-PCR. The major metabolites of *P. amarus* were qualitatively and quantitatively analyzed in the extract by using validated reversed-phase high performance liquid chromatography (HPLC) methods.

**Results:**

*P. amarus* extract significantly inhibited the production of pro-inflammatory mediators (TNF-α, IL-1β, PGE_2_) and COX-2 protein expression in LPS-induced U937 human macrophages. *P. amarus*-pretreatment also significantly downregulated the increased mRNA transcription of pro-inflammatory markers (TNF-α, IL-1β, and COX-2) in respective LPS-induced U937 macrophages. It downregulated the phosphorylation of NF-κB (p65), IκBα, and IKKα/β and restored the degradation of IκBα, and attenuated the expression of Akt, JNK, ERK, and p38 MAPKs phosphorylation in a dose-dependent manner. *P. amarus* extract also downregulated the expression of upstream signaling molecules, TLR4 and MyD88, which play major role in activation of NF-κB, MAPK and PI3K-Akt signaling pathways. The quantitative amounts of lignans, phyllanthin, hypophyllahtin and niranthin, and polyphenols, gallic acid, geraniin, corilagin, and ellagic acid in the extract were determined by HPLC analysis.

**Conclusion:**

The study revealed that *P. amarus* targeted the NF-κB, MAPK and PI3K-Akt signaling pathways to exert its anti- inflammatory effects by downregulating the prospective inflammatory signaling mediators.

**Electronic supplementary material:**

The online version of this article (10.1186/s12906-018-2289-3) contains supplementary material, which is available to authorized users.

## Background

Inflammation is an innate immune response occurring in our body to protect against harmful chemicals and invading pathogens. During inflammation, macrophages which reside in tissues and organs of human body are activated by infectious materials or injury. The activated macrophages will produce inflammatory mediators such as nitric oxide (NO), prostaglandin E_2_ (PGE_2_), interleukin 1 beta (IL-1β) and tumor necrosis factor (TNF-α), to defend against the invading pathogens [1, 2]. The occurrence of prolonged inflammatory response will lead to the development of various chronic diseases such as rheumatoid arthritis, chronic hepatitis, atherosclerosis, cancer, and inflammatory brain diseases [3–5].

Lipopolysaccharide (LPS) is a bacterial endotoxin, which activates the TLR4 receptors on macrophage and stimulates the recruitment of cytoplasmic MyD88 and TRIF adaptor proteins. The binding of the adaptor proteins on TLR4 complex will trigger the activation of nuclear factor-κB (NF-κB) and mitogen activated protein kinase (MAPK) pathways [2]. However, NF-κB is an ubiquitous nuclear transcription factor, regulates the expression of various genes which play pivotal roles in inflammation, autoimmune diseases, apoptosis and carcinogenesis [6]. In a resting cell, the NF-κB which consists of p50/p65 heterodimer bound to the inhibitory protein IκBs and remain inactive in cytoplasm. In response to LPS stimulation, activated IκB kinases (IKKs) will phosphorylate two specific serine residues (Ser32 and Ser36) on IκBs leading to its degradation through the ubiquitination and proteolysis by the 26S proteasome [2, 7, 8]. The resulting free NF-κB in cytoplasm will translocate into nucleus and binds to the specific IκB binding sites in the promoter region of target genes, followed by the transcription of various inflammatory mediators [1, 9, 10]. On the other hand, the MAPKs family consists of three major classes, extracellular signal-regulated kinases 1 and 2 (ERK1/2), c-Jun N-terminal kinase (JNK) and p38. The phosphorylation of the MAPKs in LPS-induced macrophages will trigger the transcriptional activation of NF-κB [2]. Both NF-κB and MAPK pathways will work together to aggravate the inflammatory diseases in LPS-induced inflammation models [11, 12]. The uncontrolled activation of NF-κB and MAPK signaling pathways will cause detrimental effects to the living organisms. Recent studies have found that the phosphatidylinositol 3-kinase/Akt (PI3K-Akt) signaling pathway was responsible for the expression of pro-inflammatory markers through the IκB degradation and NF-κB activation in LPS induced cells. Hence, targeting these signaling pathways will be an attractive therapeutic approach for the development of anti-inflammatory drugs [13].

Currently, non-steroidal anti-inflammatory drugs (NSAIDs) such as aspirin and ibuprofen are available to treat inflammatory diseases by inhibiting cyclooxygenase-2(COX-2) activation. However, they possess adverse effects, which include disturbance in upper gastrointestinal system and heartburn which limit their use [14]. Therefore, many researchers are trying to find a drug with greater efficacy and minimal toxicity to treat the inflammatory related diseases [15]. *Phyllanthus amarus* Schum. & Thonn. (Family: Euphorbiaceae) is a medicinal herb which is widely distributed in tropical and subtropical countries from Africa to Asia, South America and the West Indies [16]. *P. amarus* has been reported to have an array of ethanopharmacological activities such as anti-inflammatory, hepatoprotective, nephroprotective, anti-amnesia, anti-cancer, diuretic, anti-oxidant, anti-viral, anti-bacterial, anti-hyperglycemic, anti-hypercholesterolemia and so on [17–19]. The plant is a rich source of secondary metabolites such as alkaloids, flavonoids, hydrolysable tannins, lignans, polyphenols, triterpenes, sterols and volatile oils [20–23]. The anti-inflammatory activities of *P. amarus* have been demonstrated in rat models of carrageenan-induced rat paw edema air-pouch inflammation and cotton pellet granuloma [24]. Several lignans isolated from *P. amarus* such as niranthin, nirtetralin and phyltetralin exhibited in vitro and in vivo anti-inflammatory activities [25]. Previously we have reported the in vitro inhibitory effects of *P. amarus* and its isolates on phagocytic activity of human neutrophils and NO production, lymphocyte proliferation and cytokine release from phagocytes [26, 27]. Immunosuppressive effects of the standardized extract of *P. amarus* on cellular and humoral immune responses in Balb/C mice and Wistar-Kyoto rats have also been investigated [28, 29]. Despite its various pharmacological activities, there is no comprehensive investigation on molecular mechanisms underlying the anti-inflammatory effects of *P. amarus* extract in human macrophages. Hence, the present study was conducted to investigate the effects of 80% ethanolic extract of *P. amarus* on the production of pro-inflammatory mediators and the activation of signaling molecules related to NF-κB, MAPK and PI3K-Akt signaling pathways.

## Methods

### Chemicals and reagents

Roswell Park Memorial Institute (RPMI) 1640 medium, penicillin-streptomycin (Pen Strep), fetal bovine serum (FBS) were purchased from Gibco (Grand Island, NY, USA). Phorbol 12-myristate 13-acetate (PMA), LPS (*Escherichia coli* 055:B5), RIPA buffer, DMSO were purchased from Sigma Chemical Co. (St. Louis, MO, USA). 1× Halt Protease and Phosphatase Inhibitor Cocktail was purchased from Pierce (Rockford, IL, USA). Human TNF-α and IL-1β enzyme-linked immunosorbent assay (ELISA) kits were purchased from R&D Systems (Minneapolis, MN, USA). Alamar blue reagent for cell viability assay was purchased from Life Technologies (Grand Island, NY, USA). Primary antibodies specific to COX-2, p-p38, p38, p-ERK1/2, ERK1/2, p-JNK1/2, JNK1/2, p-IκBᾳ, IκBᾳ, p-IKKᾳ/β, p-NFκBp65 and β-actin were purchased from Cell Signaling Technology (Beverly, MA) and, in addition, anti-rabbit secondary antibody conjugated to horseradish peroxidase was obtained from Cell Signaling Technology (Beverly, MA). Methanol and acetonitrile of HPLC grade were purchased from Fisher Scientific (Loughborough, UK). Phyllanthin, hypophyllanthin, niranthin gallic acid, ellagic acid, corilagin and geraniin (purity > 98%) were purchased from ChromaDex (CA, USA). Dexamethasone was obtained from CCM Duopharma Biotech Bhd (Selangor, Malaysia).

### Preparation of *P. amarus* ethanol extract

The whole plants of *P. amarus* were collected from Marang, Kuala Terengganu, Malaysia, in February 2015. The plant was authenticated by Dr. Abdul Latif Mohamad of Faculty of Science and Technology, Universiti Kebangsaan Malaysia (UKM). A voucher specimen (voucher number UKMB 30075) has been deposited at the Herbarium of UKM, Bangi, Malaysia for future reference. The plant materials were dried at room temperature and powdered. The powdered plant material was macerated with 80% ethanol for 72 h and the crude ethanol extract was filtered through Whatmann No 1 filter paper. The filtrate was solvent-evaporated using rotary evaporator, freeze dried and stored in an airtight container for further investigation.

### High performance liquid chromatography analysis

Qualitative and quantitative high performance liquid chromatography (HPLC) analysis of the 80% ethanolic extract of *P. amarus* was carried out according to the method of Jantan et al. with slight modification [27]. These stock solutions (20 mg/mL) were sonicated for 15 min and filtered through 0.45 μm Millipore Millex PTFE membranes (Maidstone, Kent, UK). The solutions for reference standards (phyllanthin, hypophyllanthin, niranthin, gallic acid, ellagic acid, corilagin and geraniin) were prepared at a concentration of 1 mg/mL and further diluted into a series of concentration (1000–125 μg/mL). The HPLC analysis was performed on a Waters 2535 Quaternary Gradient Module equipped with PDA Photodiode Array Detector (Waters 2998) of wavelength ranging from 205 to 270 nm and data were acquired by using Empower 3 software. The chromatographic analysis was performed on an XBridge™ C-18 (250 mm length × 4.6 mm i.d., 5 μm) analytical column (Waters, Milford, MA, USA). Analysis of the extracts and standard solutions of lignans (phyllanthin, hypophyllanthin, niranthin) followed the following parameter: isocratic elution with solvent A. acetonitrile: solvent B. water (acidified with 0.1% orthophosphoric acid) (55:45) as mobile phase at a flow rate of 1.0 mL/min. The column was maintained at 25 °C and the detection wavelength was 205 nM. The identification and quantification of the polyphenols (geraniin, corilagin, ellagic acid and gallic acid) in the 80% ethanolic extract of *P. amarus* were carried out based on the chromatographic condition as follows: gradient elution with acetonitrile and 0.2% orthophosphoric acid as mobile phase at a flow rate of 1.0 mL/min. Quantification of compounds in the extracts was based on the standard curves equations obtained by plotting calibration curves of five concentrations (1000–125 μg/mL) each of the standard solution of compounds versus the areas under the peaks.

### Validation procedures for HPLC analysis

The HPLC method was validated by determining the linearity, precision, limits of quantification (LOQ) and detection (LOD). The precision of the method was determined by studying intra-day and inter-day variations. Separately one concentration of extracts (20 mg/mL) and reference compounds (125, 500, 1000 μg/mL) were injected three times for each concentration in one day and on three different days. The calibration curve was obtained by using phyllanthin, hypophyllanthin and niranthin as external standards. Six concentrations of each standard (31.25–1000 μg/mL) were injected in triplicate, and the curve was constructed by plotting the corresponding peak areas versus the concentration of each standard. The linearity was evaluated by linear calibration analysis while the correlation coefficient (*R*^2^) was calculated from the calibration curves. LOD and LOQ were calculated from RSD and slope (S) of the calibration curves by using following equations: LOD =3.3 × (RSD/S) and LOQ =10 × (RSD/S).

### LC-MS analysis

LC-MS analysis was performed onThermo Scientific C18 column (AcclaimTM Polar Advantage II, 3 × 150 mm, 3 μm particle size) on an UltiMate 3000 UHPLC system (Dionex). The LC-MS was carried out by using the gradient program at 0.4 mL/min, 40 °C using H_2_O + 0.1% Formic Acid (A) and 100% ACN (B) with 22 min total run time with sample injection volume of 1 uL. Gradient started at 5% B (0-3 min); 80% B (3-10 min); 80% B (10-15 min) and 5% B (15–22 min). The positive and negative ionization spectra obtained with MicroTOF QIII Bruker Daltonic with the following settings:- capillary voltage: 4500 V; nebulizer pressure: 1.2 bar; drying gas: 8 L/min at 200 °C. The mass range was at 50–1000 m/z. The accurate mass data of the molecular ions, provided by the TOF analyzer, were processed by Compass Data Analysis software (Bruker Daltonik GmbH). The corresponding peaks of the compounds were identified by comparison with the mass spectral library.

### Cell culture and differentiation induction

U937 (ATCC ® CRL-1593.2) cell line was obtained from ATCC (American Type Culture Collection). U937 mononuclear cell line was grown in RPMI 1640 medium supplemented with 10% (*v*/v) fetal bovine serum (FBS) and 1% (v/v) penicillin G/streptomycin at 37 °C under 5% CO_2._ The U937 cells density was maintained between 1 X 10^5^ and 2 X 10^6^ viable cells/mL throughout the experiments. Cells were harvested once the cell confluency reached approximately 80–90%. For all the experiments, the U937 cells were differentiated to obtain macrophage like phenotype by addition of phorbol 12-myristate 13-acetate (PMA) (Sigma-Aldrich) at 200 nM, for 24 h. The following day, cells were washed with complete culture media once and incubated overnight with serum free media for recovery phase [1, 30].

### Alamar blue for testing cell viability

The cell viability assay was carried out with Alamar blue reagent according to the manufacturer standard protocol to determine the cytotoxicity effect induced by the 80% ethanolic extract of *P. amarus*. The differentiated macrophages were plated at a density 5 × 10^5^ cells/mL onto 96 well plate. *P. amarus* extract was dissolved in DMSO, and the DMSO concentration did not exceed 1%. The cells were treated with various concentrations of *P. amarus* extract of serial dilutions 60, 30, 15, 7.5, and 3.25 μg/mL and then incubated for 24 h. After 24 h of incubation, 10% *v*/v of 10 x Alamar blue cell viability reagents was added into each well followed by 4 h incubation at 37 °C, 5% CO_2_ incubator. Then the reduction of an active compound of Alamar blue, resazurin into resorufin by viable cells was read with Tecan plate reader at 570 nm using 600 nm (normalized to 600 nm value) as a reference wavelength. The results were expressed as percentage of viable cells over control cells [19].

### Enzyme-linked immunosorbent assay

To investigate the effect of *P. amarus* extract on cytokines levels from LPS-induced cells, differentiated U937 macrophages (5 × 10^5^ cells/mL) seeded into 24 well plate were pretreated with 60, 30,15,7.5 and 3.75 μg/mL of *P. amarus* extract or with 4, 0.4, 0.04, 0.004 and 0.0004 μg/mL of dexamethasone for 2 h prior to 24 h stimulation with 1 μg/mL LPS. In another experiment, the differentiated cells also pretreated with SB202190 (a p38 inhibitor, 10 μM), U0126 (an ERK inhibitor, 10 μM), SP600125 (a JNK inhibitor, 10 μM), BAY 11–7082 (an NF-κB inhibitor, 10 μM) and LY294002 (an Akt inhibitor, 10 μM) for 2 h and then cultured with LPS of 1 μg/mL for 24 h to study the effect of inhibitors on TNF-α release. The U937 macrophages untreated with LPS which acted as a control was included for comparison. After 24 h, the cell free supernatants were collected and stored at − 20 °C until cytokine analysis. The concentrations of TNF- α and IL-1β in the supernatants of U937 cell cultures were determined using DuoSet® ELISA Development System (R&D Systems, Minneapolis, MN, USA) kit according to the manufacturer protocol [1, 19].

### Measurement of PGE_2_

The differentiated U937 cells were plated in a 24 well plate and pretreated with concentrations of *P. amarus* (60, 30 and 15 μg/mL) for 2 h and then stimulated with 1 μg/mL of LPS for 24 h. The supernatants were assayed to determine the PGE_2_ level. The PGE_2_ level was analyzed by using ELISA kit (R&D Systems, Minneapolis, MN, USA) according to manufactures protocol [1, 19].

### Quantification of relative gene expression level by qRT-PCR

U937 macrophages (1 × 10^6^ cells/mL) were pre-treated with varying concentrations of *P. amarus* (60, 30, and 15 μg/mL) for 2 h and later cultured with LPS of 1 μg/mL for 1 day. The inhibitory properties of *P. amarus* on the expression of COX-2, TNF-α, and IL-1β were evaluated by qRT-PCR [1, 19]. To determine the gene expression level, total RNA was extracted from LPS treated U937 cells by using innuPREP RNAmini kit (Analytik Jena AG, Germany) and cDNA was synthesized by using SensiFAST™ cDNA Synthesis Kit (Bioline USA Inc., Taunton, MA) following manufacturer protocol. Quantification of mRNA by qRT-PCR was done using CFX96 Touch™ Real-Time PCR Detection System (Biorad, Hercules, California, USA) along with SYBR® Green RT-PCR Master Mix (Bioline USA Inc., Taunton, MA). The cDNA was amplified by using the following primers; COX-2 (Hs_PTGS2_1_SG QuantiTect Primer QT00040586), TNF-α (Hs_TNF_3_SG QuantiTect Primer QT01079561), IL-1β (Hs_IL1B_1_SG QuantiTect Primer QT00021385) and GADPH (Hs_GAPDH_1_SG QuantiTect Primer QT00079247). The PCR reaction mixture consisted of 10 μL of SYBR master mixture, 2 μL of reverse and forward primers, 6 μL of deionized water and 2 μL of cDNA. The reaction was carried out in the following parameter: 95 °C for 2 s, 95 °C for 5 min, 60 °C for 10 min, 72 °C for 20 min (36 cycles). The relative fold difference between samples was determined following the comparative cycle threshold (2^-∆∆Ct^) method. Glyceraldehyde 3-phosphate dehydrogenase (GADPH) was used as the housekeeping gene for normalizing the data.

### Western blotting analysis

Differentiated cells (1 × 10^6^ cells/mL) were plated onto six well plate and pretreated with 60, 30, 15 μg/mL of *P. amarus* extract, SB202190, U0126, SP600125, LY294002 and BAY 11–7082 (10 μM) for 2 h and then stimulated with 1 μg/mL of LPS for 30 min. The cells were harvested by centrifugation at 7000 x *g* for 5 min and washed twice with ice cold PBS. The washed cell pellets were lysed in RIPA buffer and halt protease and phosphatase inhibitor cocktails. The cell lysates were centrifuged at 13,000 x *g* for 10 min and protein concentrations were measured by Bradford assay. Approximately 20 μg of proteins were resolved by 10% SDS-polyacrylamide gel electrophoresis and were electroblotted onto polyvinylidene difluoride (PVDF) membrane. The immunoblot was incubated for 1 h with 5% skim milk powder in TBS-T buffer containing 0.1% Tween 20 to block the nonspecific binding prior to the overnight incubation with specific primary antibodies that recognized COX-2, p-p38 (Thr180/Tyr182), p38, p-ERK1/2 (Thr202/Tyr204), ERK1/2, p-JNK1/2 (Thr183/Tyr185), JNK1/2, p-IκBα (Ser32/36), IκBα, p-IKKα/β (Ser176/180), p-NFκBp65 (Ser536), p-Akt (Ser 473) and β-actin (Cell Signaling Technology Inc., Beverly, MA). The following day, membrane was washed with TBS-T (0.1% Tween 20) 3 times for 10 min each. It was then incubated with anti-rabbit secondary antibody conjugated to horseradish peroxidase for 1 h at room temperature with agitation and washed 3 times with TBST for 10 min each. Each protein band was detected using chemiluminescence detection system according to the manufacturer instruction. The band intensity was quantified using Image Lab™ software [1, 2, 31].

### Statistical analysis

Statistical analyses were performed using the GraphPad Prism 6.0 (GraphPad Software, San Diego, CA, USA). For each experiment, three independent experiments were performed and data are expressed as mean ± standard error mean (SEM). Differences between two means were analyzed by one-way analysis of variance (ANOVA) followed by post-hoc Dunnett’s test with *P* < 0.05 considered as statistically significant.

## Results

### Quantitative and qualitative analysis of 80% ethanolic extract of *P. amarus*

**Fig. 1:**
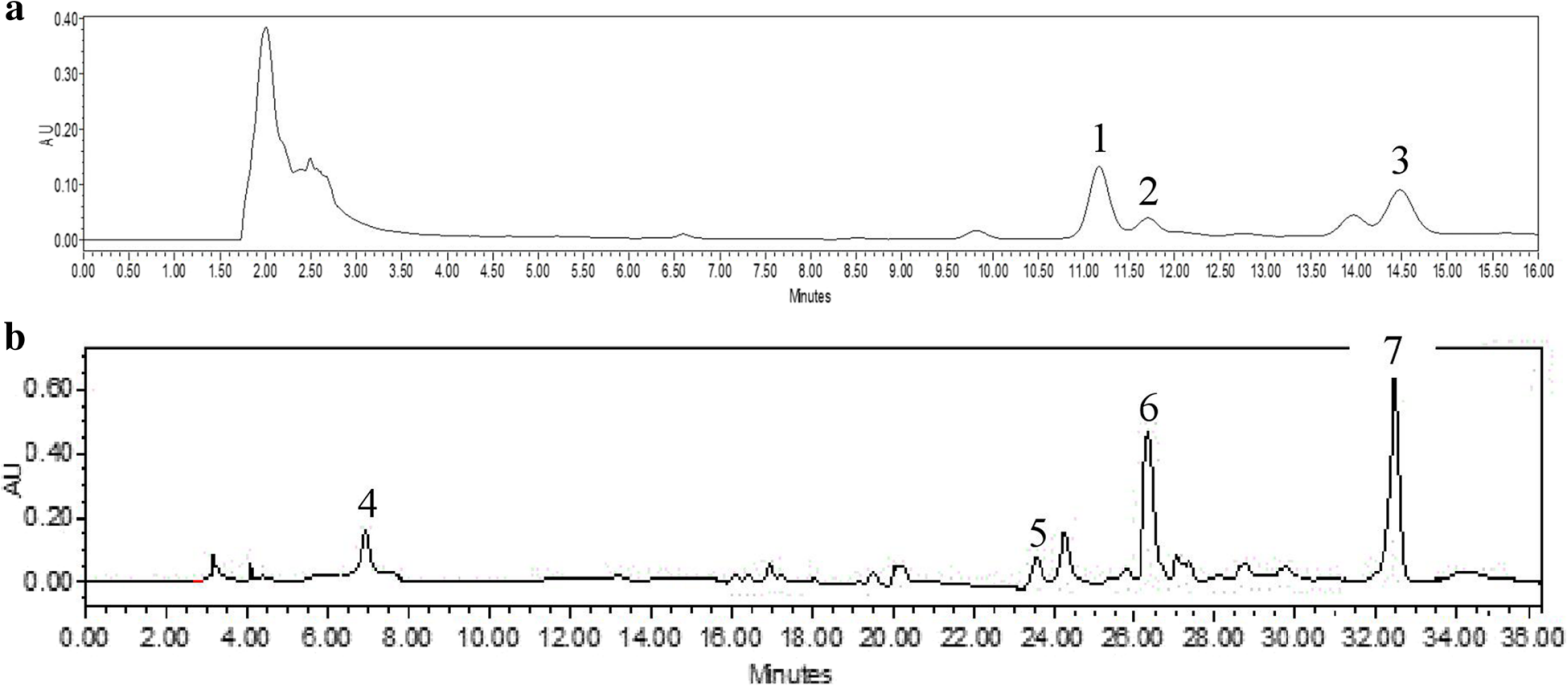
RP-HPLC chromatogram of the 80% ethanol extracts of *Phyllanthus amarus* **a** for identification and quantification of (1) phyllanthin, (2) hypophyllanthin and (3) niranthin; **b** for identification and quantification of (4) gallic acid, (5) geraniin, (6) corilagin and (7) ellagic acid at the wavelength of 205 nm

To identify and quantify the active components of *P. amarus* we performed HPLC analysis of the ethanol extract and several representative standards as previously reported by Jantan et al. [32]. Peaks with the same retention time as the standard compounds, phyllanthin (11.17 min), hypophyllahtin (11.71 min) and niranthin (14.48 min) were observed in *P. amarus* (Fig. 1a). For the polyphenols, four peaks were identified namely gallic acid (7.89 min), geraniin (23.76 min), corilagin (26.41 min) and ellagic acid (33.42 min) (Fig. 1b). Among the lignans, phyllanthin (660.28 μg/mL) was present at the highest concentration compared to niranthin (575.11 μg/mL) and hypophyllanthin (290.46 μg/mL). Among the identified polyphenols, ellagic acid (601.29 μg/mL) was the most abundant, followed by corilagin (313.41 μg/mL), geraniin (170.49 μg/mL) and gallic acid (163.30 μg/mL). The LC-MS results revealed that *P. amarus* extract consisted of 18 compounds identified via positive ionization mode while five compounds identified via negative ionization mode (Table 1).

**Table 1:** Tentative compounds detected in 80% ethanol extract of *Phyllanthus amarus* by LCMS analysis

Peak	Retention time (RT)	Molecular ion peak	MS^2^ fragment ions	Tentative compounds identified
1	1.7	131 (M-H)^−^	909, 688, 244, 134	D-glutamine
2	2.0	195 (M-H)^−^	909, 678, 404, 204	Gluconic acid
3	2.1	228 (M-H)^−^	897, 709, 405, 238, 129	Naringenin
4	2.1	191 (M-H)^−^	935, 596, 381, 191	Quinic acid
5	2.6	133 (M-H)^−^	913, 677, 412, 230, 134	D-(+)-Malic acid
6	2.9	321 (M-H)^−^	711, 412, 248, 183	Dinitramin
7	3.1	128 (M-H)^−^	122	4-oxoproline
8	4.2	387 (M-H)^−^	514, 387, 207	galloyl glucopyroxidase
9	6.5	169 (M-H)^−^	169	Gallic acid
10	7.0	343 (M-H)^−^	169	trio-O-methylellagic acid
11	7.9	633 (M-H)^−^	633, 463, 301	corilagin
12	8.0	331 (M-H)^−^	169	tuberonic acid hexoside
13	8.5	951 (M-H)^−^	933, 765, 613, 463, 301	geraniin
14	8.9	965 (M-H)^−^	933, 765, 609, 300	Rutin
15	9.2	197 (M-H)^−^	125	Syringic acid
16	9.2	463 (M-H)^−^	463, 300, 169	Quercetine 3-D-glucoside
17	9.5	206 (M-H)^−^	873, 704, 558, 226, 147	N-acetyl-D- phenylalanine
18	9.7	247 (M-H)^−^	247	7-Deshydroxypyrogallin4- carboxylic acid
19	1.6	133 (M-H)^+^	663, 383, 132	Maleamate
20	1.8	222 (M-H)^+^	222	Metaxalone
21	1.9	184 (M-H)^+^	184	4-pyridoxic acid
22	3.6	166 (M-H)^+^	120	Stachydrine
23	13.9	419 (M-H)^+^	419, 149	Phyllanthin

### Effects of *P. amarus* on cell viability

U937 macrophages were incubated with the 80% ethanolic extract of *P. amarus* ranging from 0 to 60 μg/mL and cell viability was determined by alamar blue assay after 24 h. The results demonstrated that from 0 to 60 μg/mL of *P. amarus* extract, there was no cytotoxic effect on U937 macrophages (cell viability greater than 90%). These results confirmed that the effects of *P. amarus* on U937 macrophages in this study were not due to cytotoxicity.

### Effects of *P. amarus* on pro-inflammatory cytokines production and gene expression

**Fig. 2:**
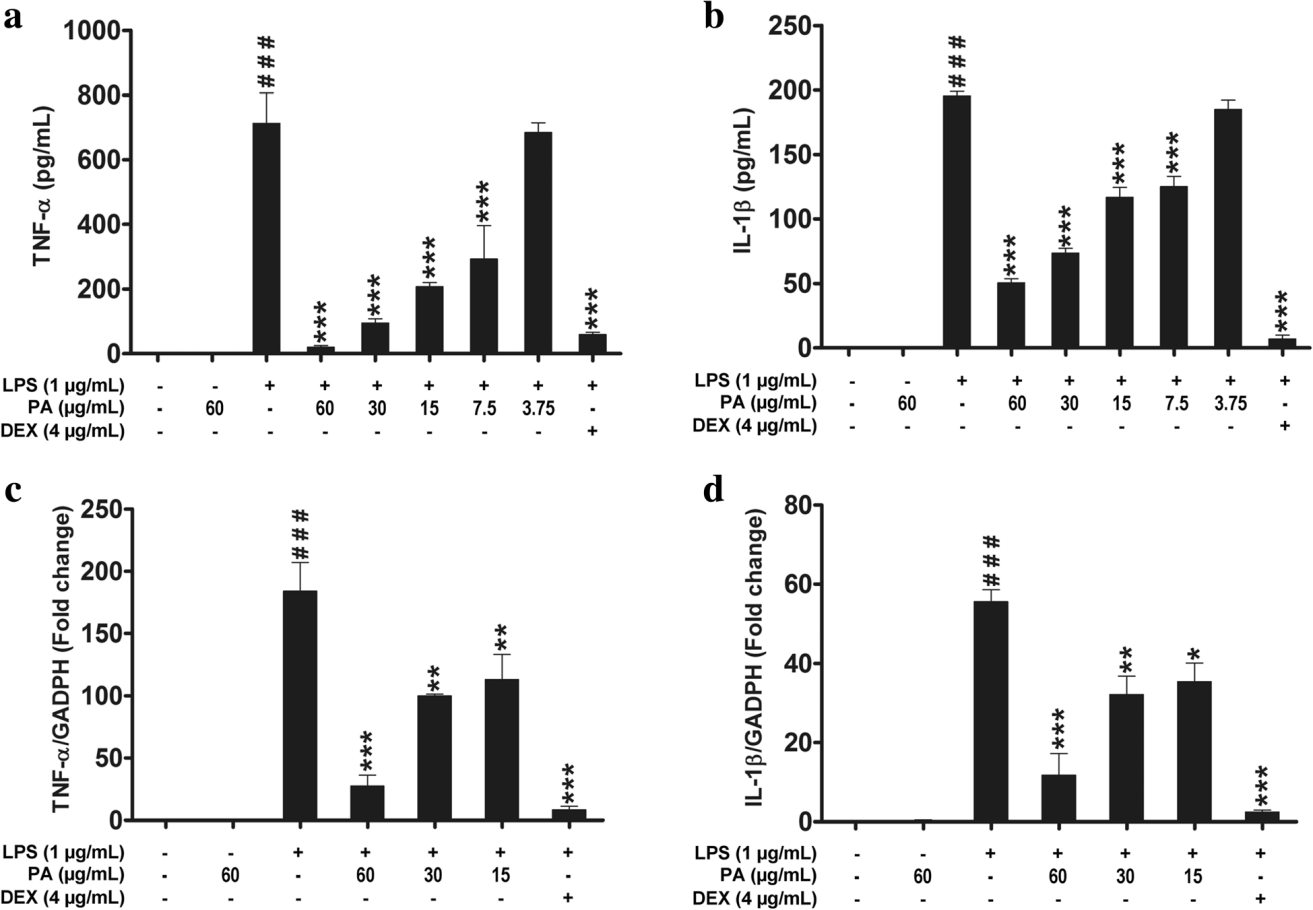
Effects of *Phyllanthus amarus* on the release of pro-inflammatory cytokines **a** TNF-α **b** IL-1β. Effects of *P. amarus* on the mRNA expression of **c** TNF-α **d** IL-1β. Data are presented as mean ± SEM (*n* = 3). ^###^*P* < 0.001 represents the significant difference from the control. *P < 0.05, **P < 0.01, and ***P < 0.001 represent significance to the LPS alone versus PA or DEX pretreated

To investigate the anti-inflammatory effects of *P. amarus* extract on LPS-stimulated macrophages, firstly the concentrations of TNF-α and IL-1β in the culture supernatants of U937 macrophages were determined by ELISA kit. The results demonstrated that, the pro- inflammatory cytokines (TNF-α and IL-1β) production was significantly upregulated in LPS induced U937 macrophages while the cells pretreated with *P. amarus* for 2 h prior to LPS stimulation showed suppressive effect on TNF-α and IL-1β production with IC_50_ values of 16.12 and 7.13 μg/mL, respectively (Fig. 2a and b). In addition, the suppressive effect also was observed for sample treated with an standard anti-inflammatory agent, dexamethasone which exhibited inhibitory activity on TNF-α and IL-1β with IC_50_ values of 0.18 μg/mL and 0.002 μg/mL, respectively. Next, we determined the effects of *P. amarus* on pro-inflammatory cytokines expression at pre-translational level by using quantitative Real-Time RT-PCR (qRT-PCR). As shown in Additional file 1: Figure S1, the mRNA expression of TNF-α and IL-1β were significantly (*P* < 0.001) upregulated in U937 macrophages 24 h following LPS stimulation. However, the cells pretreated with *P. amarus* (60, 30 and 15 μg/mL) for 2 h showed a significant inhibition in LPS induced TNF-α (27, 32, and 35 fold, respectively) and IL-1β (12, 32, and 35 fold, respectively) (Fig. 2c and d). These gene expression levels also were comparable with 4 μg/mL dose of dexamethasone pretreated cells prior to LPS induction. Dexamethasone pretreated cells also showed an inhibition of TNF-α and IL-1β expression (8 and 2 fold respectively) (Fig. 2c and d). The consistent inhibition of cytokine expression at protein and mRNA levels suggests that *P. amarus* may exert their anti-inflammatory effects by controlling gene transcription.

### Effects of *P. amarus* on PGE_2_ production and COX-2 expression

**Fig. 3:**
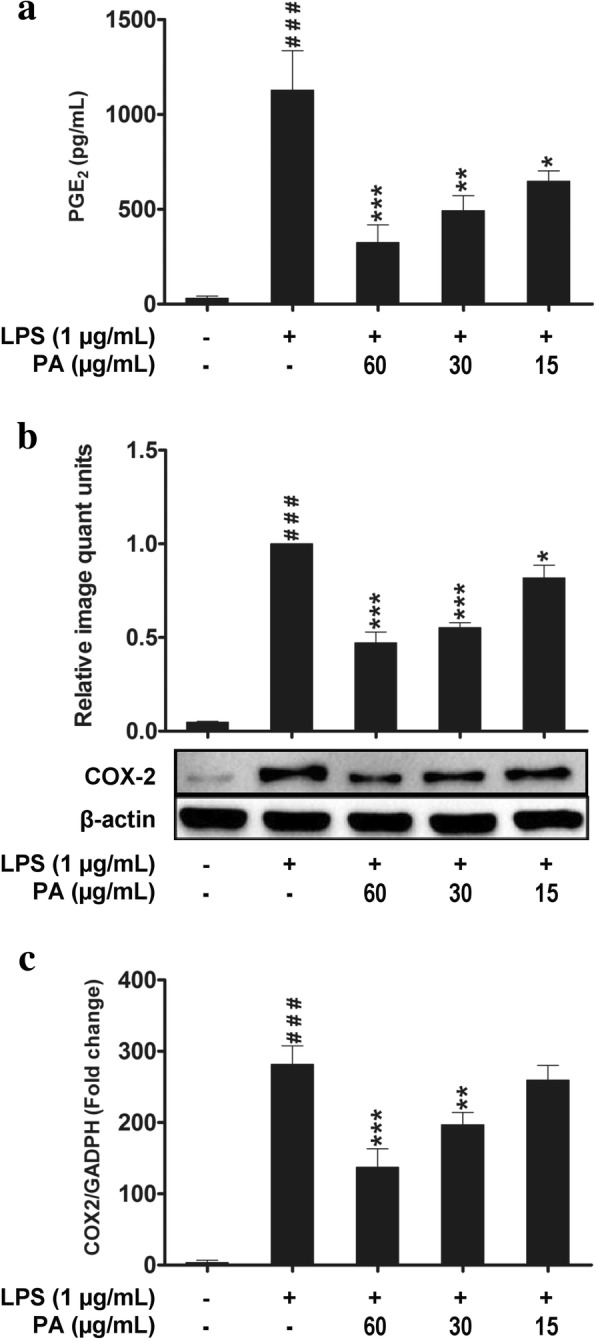
Effects of *Phyllanthus amarus* on the release of PGE_2_ production (**a**). Effects of *P. amarus* on COX-2 protein expression (**b**) and mRNA expression (**c**). Data are presented as mean ± SEM with (*n* = 3). ^###^*P* < 0.001 represents the significant difference from the control. *P < 0.05, **P < 0.01, and ***P < 0.001 represent significance to the LPS alone versus PA

To examine the effect of *P. amarus* on PGE_2_ release, production of PGE_2_ in culture supernatant was measured. PGE_2_ production was measured in U937 macrophages induced with LPS for 24 h in the presence and absence of *P. amarus*. As shown in Fig. 3a, LPS (1 μg/mL) induced U937 macrophages produced significant amount of PGE_2_ (1128.67 ± 200.07 pg/mL) compared to control (31.33 ± 11.31 pg/mL). However, the production of PGE_2_ was significantly attenuated by *P. amarus* pretreatment at 60, 30 and 15 μg/mL in a dose- dependent manner. In order to examine the mechanisms by which *P. amarus* inhibited LPS- induced PGE_2_ release, the expression of COX-2 at protein and gene level was measured in U937 macrophages induced with LPS. As shown in Fig. 3b, Western blot analysis showed that 24 h LPS stimulation significantly (*P* < 0.001) upregulated the COX-2 protein expression while 2 h *P. amarus* (60, 30 and 15 μg/mL) pretreatment significantly attenuated the expression in a dose-dependent manner. In addition, we have investigated the effects of *P. amarus* on gene expression to determine whether *P. amarus* inhibited the LPS induced COX-2 mRNA level in U937 macrophages. As shown in Fig. 3c, the significantly (*P* < 0.001) increased COX-2 mRNA expression (282 fold) in LPS stimulated cells were significantly attenuated in *P. amarus* (60 and 30 μg/mL) pretreated cells (137 and 259 fold, respectively) as compared to control.

### Effects of *P. amarus* on NF-κB signaling pathways

**Fig. 4:**
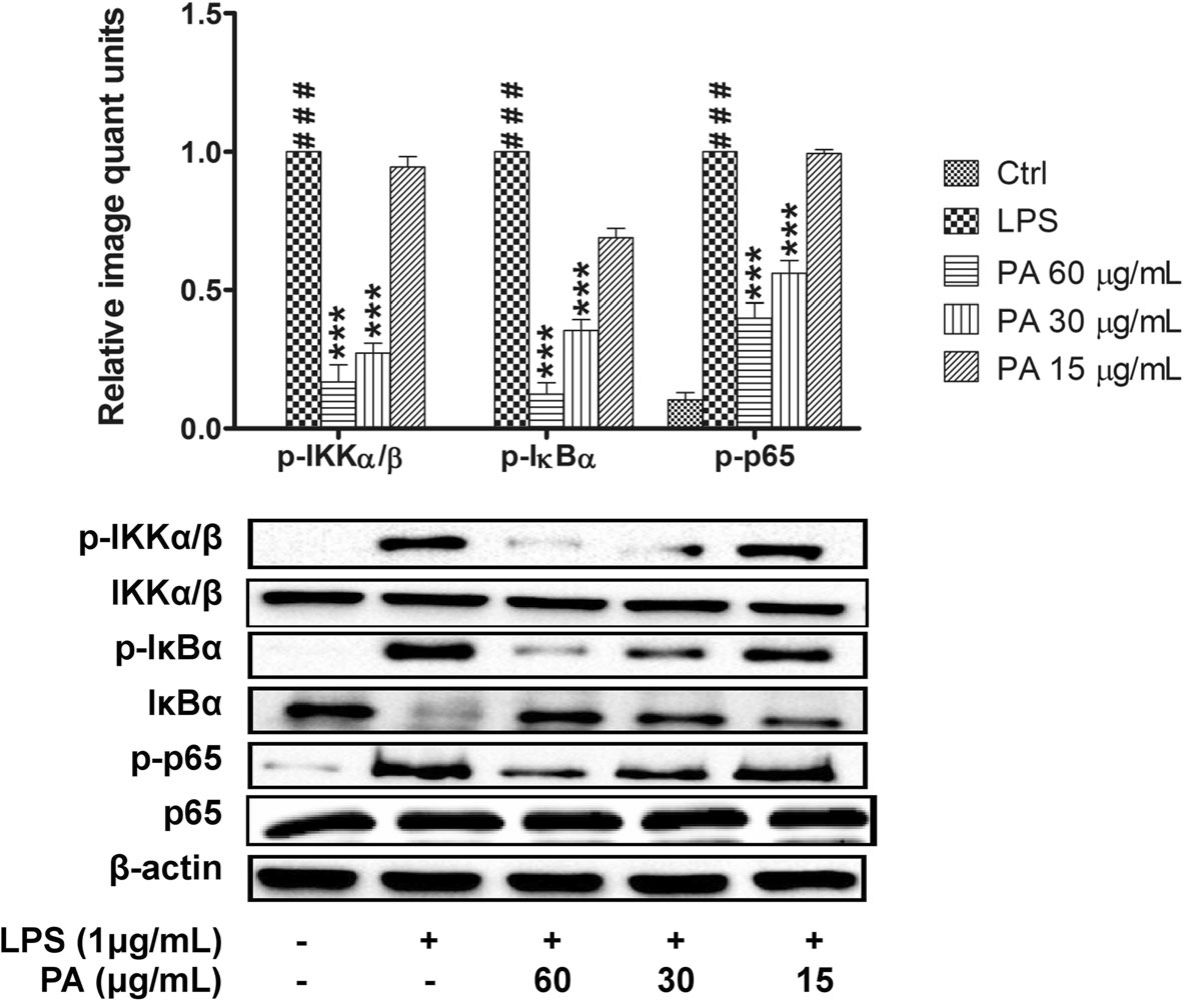
Effects of *Phyllanthus amarus* on phosphorylation of IKKα/β, IκBα and NF-κB (p65). Data are presented as mean ± SEM with (*n* = 3). ^###^*P* < 0.001 represents the significant difference from the control. *P < 0.05, **P < 0.01, and ***P < 0.001 represent significance to the LPS alone versus PA

To determine the role of IKK/IκB/NF-κB signaling pathway in *P. amarus* mediated inhibition of LPS-induced inflammatory response, we studied the activation of p-NF-κB (p65), NF-κB (p65), p-IκBα, IκBα, p-IKKα/β, and IKKα/β in LPS-induced human macrophages by Western blot. Our results demonstrated that, *P. amarus* altered the NF-κB signaling mechanisms by suppressing the phosphorylation of IKKα/β and IκBα in a dose-dependent manner, which were significantly (*P* < 0.001) upregulated by 30 min LPS treatment. In addition, the 2 h *P. amarus* pretreatment significantly blocked the degradation of LPS induced IκBα in a concentration-dependent manner (Additional file 2: Figure S2). Furthermore, the 2 h pretreatment with *P. amarus* also significantly attenuated the LPS-induced phospho-p65 without altering the total level of p65 in human U937 macrophages (Fig. 4).

### Effects of *P. amarus* on MAPKs and PI3K-Akt activation

**Fig. 5:**
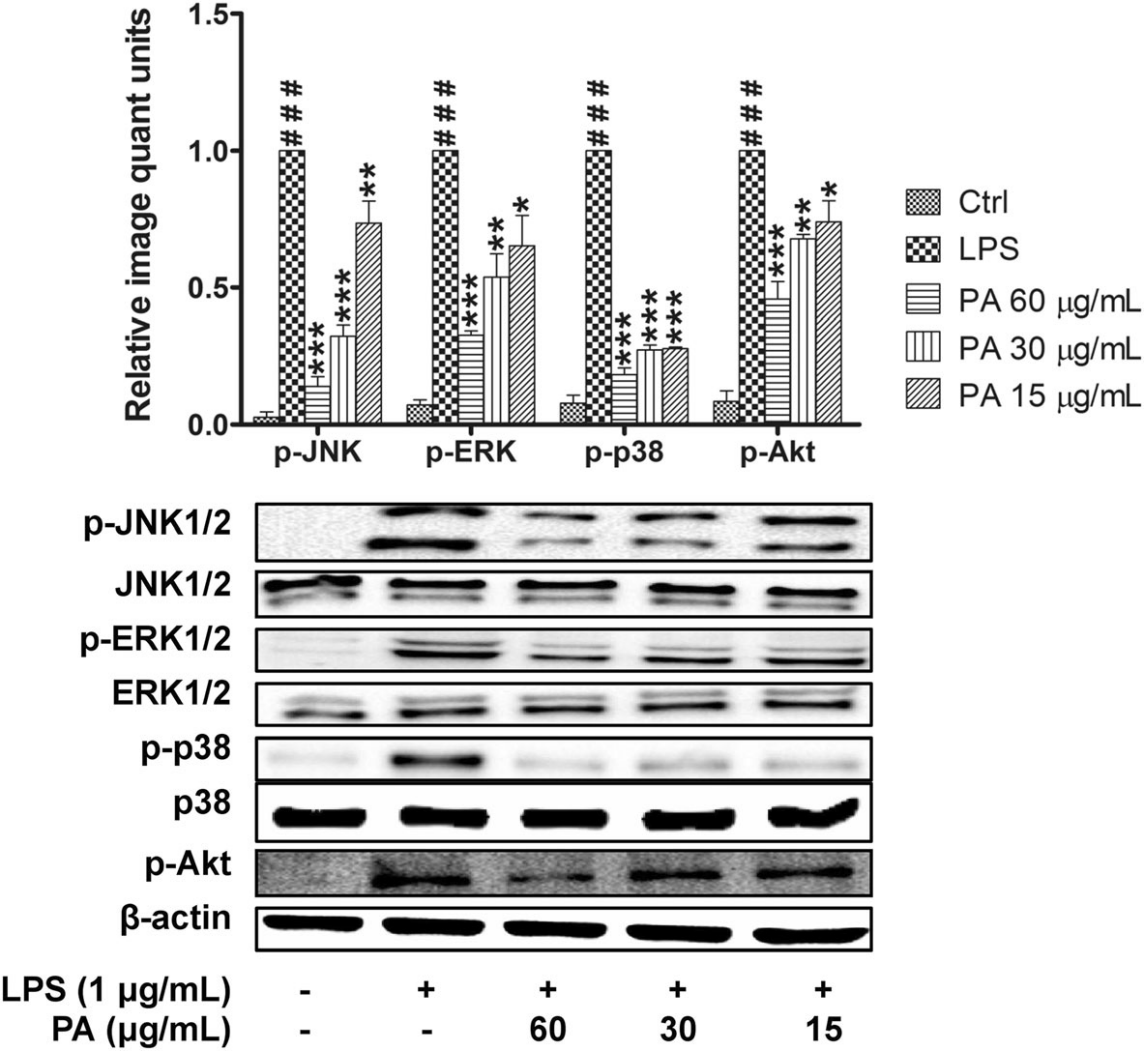
Effects of *Phyllanthus amarus* on p-JNK, p-ERK, p-p38 and p-Akt expression. Data are presented as mean ± SEM with (*n* = 3). ^###^*P* < 0.001 represents the significant difference from the control. *P < 0.05, **P < 0.01, and ***P < 0.001 represent significance to the LPS alone versus PA

Studies have demonstrated that the activation of Akt and MAPKs (JNK1/2, ERK1/2, and p38) signaling molecules is important to initiate and mediate the NF-κB signal transduction pathway. Hence,we investigated the effect of *P. amarus* on phosphorylation of Akt by Western blot analysis. As depicted in Fig. 5, the 30 min LPS induction significantly upregulated Akt phosphorylation while 2 h *P. amarus* pretreatment dose-dependently suppressed the phosphorylation. Furthermore, the effect of PA on phosphorylation activity of three important MAPKs signaling molecules (JNK1/2, ERK1/2, and p38) was also investigated. The LPS induction at 30 min significantly (*P* < 0.001) stimulated the JNK1/2, ERK1/2, and p38 phosphorylation levels in macrophages but the cells pretreated with *P. amarus* (60, 30 and 15 μg/ml) for 2 h significantly suppressed the phosphorylation of JNK1/2, ERK1/2, and p38 protein kinases in a dose-dependent manner without interfering the total level of JNK1/2, ERK1/2, and p38 protein kinases (Fig. 5).

**Fig. 6:**
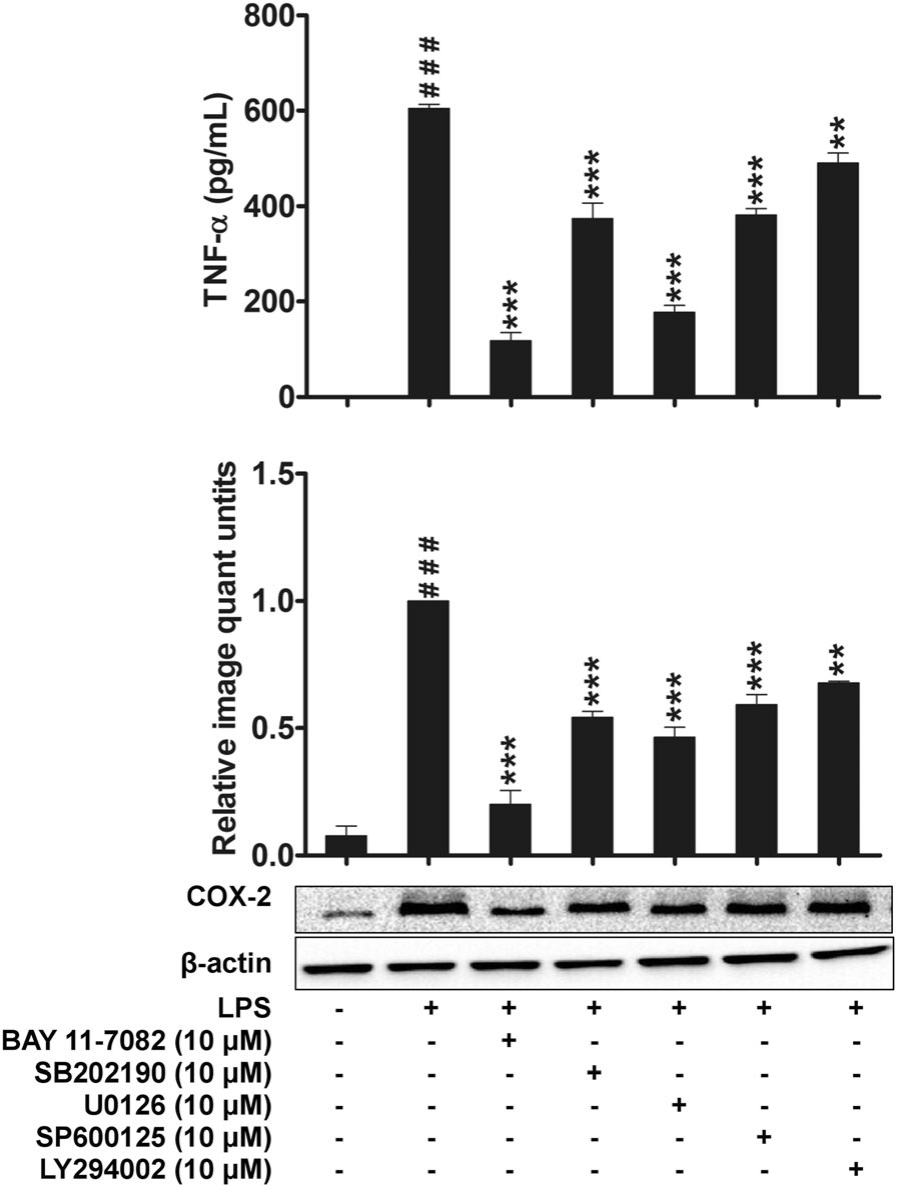
Effects of SB202190 (p38 inhibitor, 10 μM), U0126 (ERK inhibitor, 10 μM), SP600125 (JNK inhibitor, 10 μM), BAY 11–7082 (NF-κB inhibitor, 10 μM) and LY294002 (Akt inhibitor, 10 μM) on TNF-α production and COX-2 protein expression in LPS stimulated U937 macrophages. The values are expressed as the mean ± SEM with (*n* = 3). ^###^*P* < 0.001 indicates significant difference from the unstimulated control group. ***P < 0.001 indicate LPS versus LPS + inhibitors pretreatment

In order to further validate that the suppression of inflammatory mediators by *P. amarus* linked to the NF-κB, MAPKs, and Akt signaling pathway down regulation, we examined the effect of specific NF-κB, MAPKs, and Akt inhibitors on TNF-α production and COX-2 protein expression. The 2 h pretreatment of LPS-induced macrophages with BAY 11–7082 (an NF-κB inhibitor), LY294002 (an Akt inhibitor), SB202190 (a p38 inhibitor), U0126 (an ERK inhibitor) and SP600125 (a JNK inhibitor) showed significant inhibitory effect on TNF-α production and COX-2 expression (Fig. 6). Taken together, our results suggest that *P. amarus* reduced COX-2 expression and TNF-α production by down regulating LPS-activated NF-κB, ERK, JNK, p38MAPKs and Akt signaling pathways.

### Effects of *P. amarus* on MyD88 and TLR4 signaling molecules

**Fig. 7:**
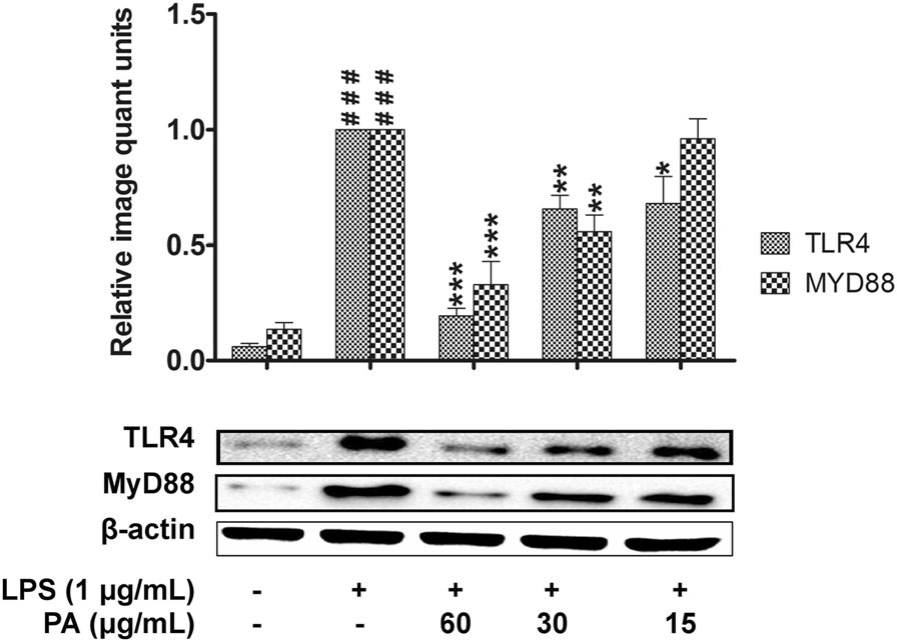
Effects of *Phyllanthus amarus* on TLR4 and MyD88 expression. Data are presented as mean ± SEM with (*n* = 3). ^###^*P* < 0.001 represents the significant difference from the control. *P < 0.05, **P < 0.01, and ***P < 0.001 represent significance to the LPS alone versus PA

To further confirm the anti- inflammatory activity of *P. amarus*, the expression of MyD88 and TLR4 were investigated in LPS-induced U937 macrophages. As shown in Fig. 7, 1 h LPS induction significantly (*P* < 0.001) upregulated the expression of MyD88 and TLR4 compared to control cells. However, the cells pretreated with *P. amarus* for 2 h dose- dependently diminished the upregulation of MyD88 and TLR4 protein expression. It was noted that, at the dose of 60 μg/mL, the suppression of *P. amarus* was found highly significant (*P* < 0.001) for the both upstream signaling molecules.

## Discussion

*P. amarus* is a promising herbal resource with therapeutic potential against various diseases due to the presence of numerous active and secondary metabolites [22]. Although *P. amarus* extracts have been previously shown to possess immunosuppressive activity on immune cells but the mechanism underlying the anti-inflammatory activity of the plant remain unknown [27, 32–34]. Therefore, in the present study we investigated the in vitro effects of *P. amarus* by using LPS-activated U937 human macrophage, which mimics the inflammatory model [1]. Macrophages can be activated by LPS to produce pro-inflammatory molecules such as IL-6, TNF-α, IL-1β, PGE_2_ and NO by triggering intracellular signaling pathways including NF-κB and MAPKs [1, 2, 35].

COX is known as an enzyme which aid in the process converting the arachidonic acid to prostaglandins and it exists as COX-1 and COX-2 isomers. COX-1 is responsible for the homeostatic function of PGE_2_ while COX-2 triggers the excess release of PGE_2_ at the region of inflammation which is known to play critical role during pathogenesis of chronic diseases [31, 36, 37]. Besides, it is also reported that overexpression of COX-2 leads to the upregulation of several angiogenic mediators [38]. Therefore, potent inhibitory effects of anti-inflammatory drugs on COX-2 expression have been revealed by several researches to be valuable in preventing and curing these disorders [39]. In current study, we found that *P. amarus* extract suppressed the PGE_2_ production in LPS stimulated U937 macrophages by down regulating the COX-2 protein and gene expression. These finding suggest that the suppression of PGE_2_ release by *P. amarus* extract might be due to the inhibition of COX-2 elevation upon the LPS induction of macrophages. Furthermore, Kiemer et al. also reported the inhibitory effect of *P. amarus* extract on PGE_2_ production in rat Kupffer cells and RAW264.7 macrophages, whick were in consistent with our present finding. Like COX-2, iNOS is also one of the enzyme that is involved in the production of excess NO during chronic inflammatory disorders to alleviate the pathogenesis of disease state [38]. It is to be noted that from our experiment NO could not be detected at measurable quantity in the LPS induced U937 macrophages which is in agreement with the previous report [40]. The probable reason for this occurrence may linked to the statement that, U937 cells lack of BH4 (tetrahydrobiopterin) which is the crucial cofactor for NO production [19, 41]. TNF-α and IL-1β are known as a key pro-inflammatory cytokines that are secreted during the development of chronic inflammatory diseases [42]. In this study we found that the extract of *P. amarus* and positive control, dexamethasone showed a significant inhibition of pro-inflammatory cytokines in a concentration-dependent manner in LPS-stimulated human macrophages. The outcome was found in line with the previous study where the inhibitory effect of *P. amarus* on pro-inflammatory cytokines such as TNF-α, IL-1β and IFN-γ in LPS induced immune cells has also been observed [32].

NF-κB signaling plays a major role in macrophages to regulate the cell survival genes and induces transcription and translation of other mediators that are involved in inflammatory response [43]. Thus, the inhibition of these signaling pathways may highlight the potential of *P. amarus* as a suppressor of inflammatory cytokines. Consistent with previous report, *P. amarus* ethanolic extract inhibited the production of LPS-induced TNF-α and IL-1β that are known to regulate by NF-κB signaling pathway. Interestingly, our present findings revealed that *P. amarus* extracts dose –dependently diminished the phosphorylation of IKKα/β and IκBα in response to LPS. Furthermore, LPS-induced IκBα degradation in U937 cells also inhibited by *P. amarus* in a dose dependent manner. These findings indicate that the downregulation of LPS-induced inflammatory cytokines production by *P. amarus* results from the blockade of IKK/IκB/NF-κB signaling pathway. A study has reported that *P. amarus* inhibited the DNA binding activity of NF-κB binding factors and also iNOS and COX-2 expression in LPS-induced murine macrophages [32]. This is in agreement with our findings which showed that *P. amarus* was able to downregulate the NF-κB activation by suppressing the release of pro- inflammatory mediators.

Apart from NF-κB, LPS-induced macrophages also activate MAPKs signaling pathway, which regulate the expression of inflammatory mediators by controlling NF-κB activity [44]. Our study demonstrated that pretreatment of cell with *P. amarus* inhibited the early activation of cell by LPS in a dose-dependent manner. Thus, our study strongly indicates that the MAPKs (JNK, ERK, and p38) were involved in inhibitory activity of *P. amarus* on the expression of pro-inflammatory mediators. Apart from NF-κB and MAPKs, phosphatidylinositol 3-kinase/Akt (PI3K-Akt) is another signaling pathway which is activated in LPS induced macrophages to control the expression of inflammatory markers by activating the NF-κB signal transduction pathway [45]. Hence, blocking the phosphorylation activity of Akt in response to LPS induction is known to be an important target to control inflammatory disorders. Supportingly, our study revealed that, *P. amarus* pretreatment significantly attenuated the LPS induced Akt phosphorylation in LPS induced human macrophages. Furthermore, in this study we proved that the suppression of TNF-α production and COX-2 protein expression by *P. amarus* were due to the blockage of NF-κB, MAPKs, and Akt pathways through the use of NF-κB, MAPKs, and Akt inhibitors.

TLR4 is known to specifically bind with LPS and trigger inflammatory response by activating NF-κB, MAPKs, and PI3K-Akt signaling pathways which lead to the production of inflammatory mediators [1, 46, 47]. The activated TLR4 sequentially transmit the inflammatory signals through the adaptor protein, Myd88 [48]. This suggests that, TLR4 and MyD88 act as a specific molecular target to inititate inflammatory responses [47, 49]. Our investigation demonstrates that *P. amarus* significantly inhibited LPS-induced TLR4 and MyD88 expression in U937 macrophages. These findings propose that TLR4 and MyD88 participated in the inhibitory action of *P. amarus* on the LPS-induced production of PGE_2_ and pro-inflammatory cytokines.

## Conclusions

In conclusion, the present study demonstrated that *P. amarus* potently suppressed the inflammatory responses in LPS-induced U937 macrophages via inhibition of MyD88-dependent signaling pathway, which may be linked to the inhibitory effects exerted by *P. amarus* on pro-inflammatory mediators. Therefore, the ethanol extract of *P. amarus* have promising anti-inflammatory activity which acts through the suppression of NF-κB, MAPKs, and PI3K-Akt signaling pathways and may have beneficial therapeutic applications for treating inflammatory disorders.

## Additional files

Additional file 1: **Figure S1.** The mRNA expression of TNF-α and IL-1β at various time points. (TIF 3977 kb)

Additional file 2: **Figure S2.** Effects of *Phyllanthus amarus* on degradation of IκBα. (TIF 97 kb)

## Abbreviation

ATCC: American type culture collection
COX2: Cyclooxygenase 2
ELISA: Enzyme-linked immunosorbant assay
ERK: Extracellular signal-regulated kinase
FBS: Fetal bovine serum
HPLC: High performance liquid chromatography
IL-1β: Interleukin 1 beta
iNOS: Inducible nitric oxide synthase
IκB: I-kappa B kinase
JNK: c-Jun N-terminal kinase
LPS: Lipopolysaccahride
MAPK: Mitogen activated protein kinase
MyD88: Myeloid differentiation primary response gene 88
NF-κB: Nuclear factor-kappa B
NO: Nitric oxide
NSAID: Non-steroidal anti-inflammatory drug
PA: *Phyllanthus amarus*
PGE_2_: Prostaglandin E2
PMA: Phorbol 12-myristate 13-acetate
RPMI: Roswell park memorial institute
TLR4: Toll like receptor 4
TNF-α: Tumor necrosis factor-alpha
