# Supplementary figures and images for: Anti-inflammatory effects of Phyllanthus amarus Schum. & Thonn. through inhibition of NF-κB, MAPK, and PI3K-Akt signaling pathways in LPS-induced human macrophages
Source: BMC Complement Altern Med. 2018 Jul 25;18:224. doi: 10.1186/s12906-018-2289-3 (PMC6060475; doi:10.1186/s12906-018-2289-3)

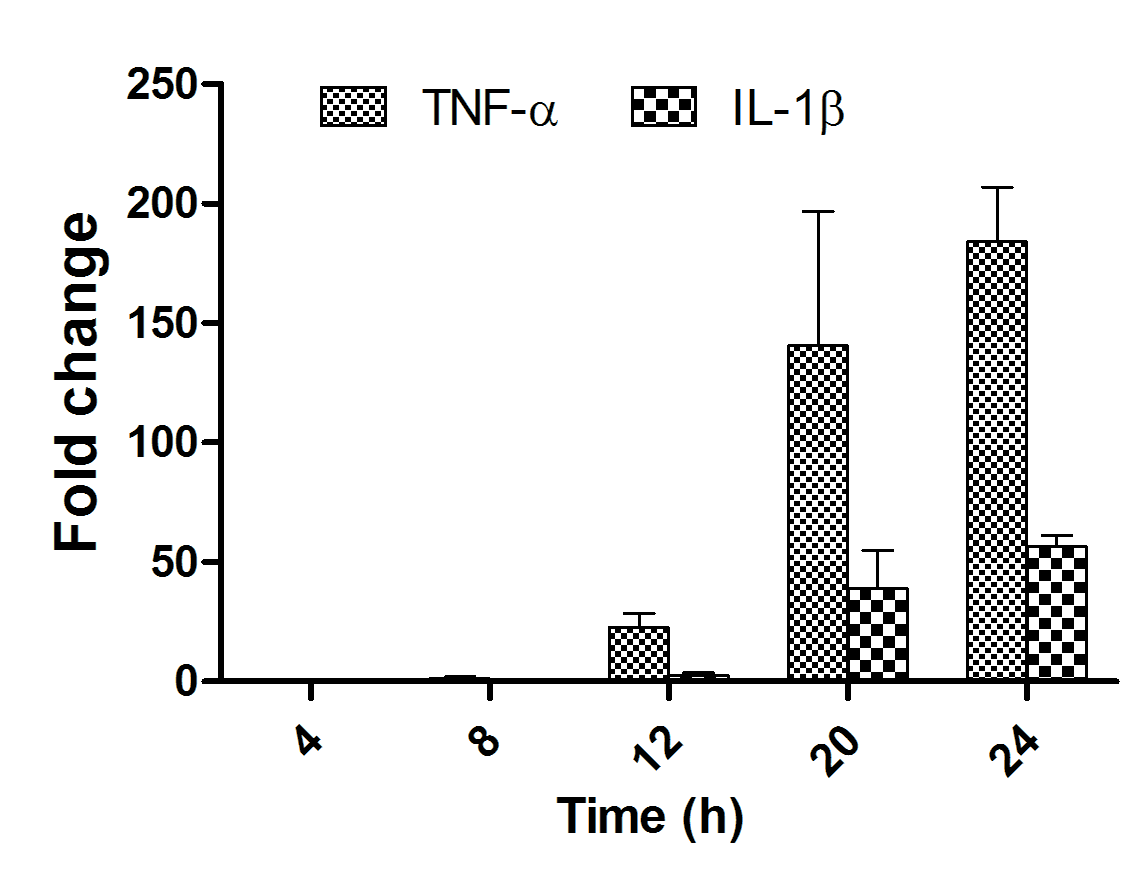

Supplement: Supplementary file 1 — Figure S1. The mRNA expression of TNF-α and IL-1β at various time points. (TIF 3977 kb) [file 12906_2018_2289_MOESM1_ESM.tif]

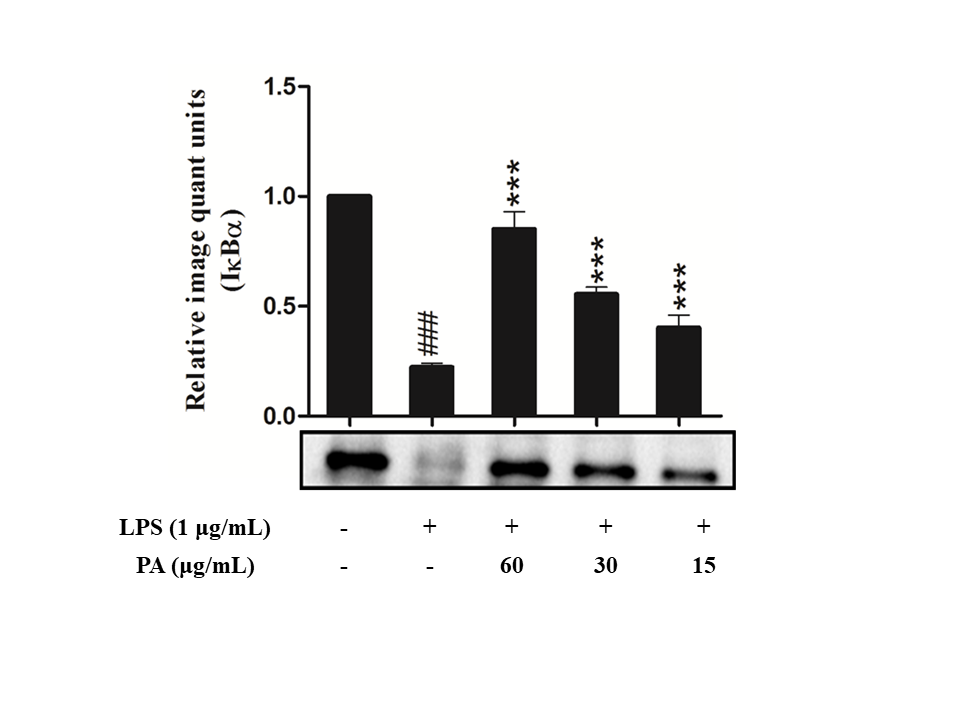

Supplement: Supplementary file 2 — Figure S2. Effects of Phyllanthus amarus on degradation of IκBα. (TIF 97 kb) [file 12906_2018_2289_MOESM2_ESM.tif]
